# Analysis of the Inhomogeneous Growth of Sputtered Black TiO_2_ Thin Films

**DOI:** 10.1021/acsomega.3c09772

**Published:** 2024-03-21

**Authors:** Dennis Berends, Patrick Schwager, Kai Gehrke, Martin Vehse, Carsten Agert

**Affiliations:** DLR Institute of Networked Energy Systems, Urban and Residential Technologies, 26129 Oldenburg, Germany

## Abstract

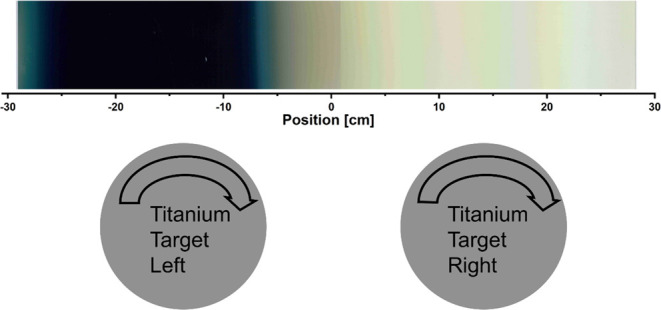

Black titanium dioxide (B-TiO_2_) is a highly active photoelectrochemical material compared to pure titanium dioxide due to its increased light absorption properties. Recently, we presented the deposition of thin-film B-TiO_2_ using an asymmetric bipolar reactive magnetron sputter process. The resulting samples exhibit excellent photoelectrochemical properties, which can be fine-tuned by varying the process parameters. In this article, results of morphological, electrical, and photoelectrochemical measurements are discussed to better understand the surprisingly high electrochemical activity of the films. In order to study the influence of the dynamic process on film formation, we use static sputtering with a fixed substrate covering the entire chamber area in front of the two targets. This allows the material composition of the sputtered film to be analyzed depending on its relative position to the targets. The results lead to the conclusion that the asymmetric bipolar sputtering mainly produces two phases, a transparent, nonconductive crystalline phase and a black, conductive amorphous phase. As a consequence, the dynamically sputtered samples are multilayers of these two materials. We discuss that the significantly better electrical and photoelectrochemical properties emerge from the inhomogeneous nature of the laminates, like also found in core–shell nanoparticles of B-TiO_2_.

## Introduction

Black titanium dioxide (B-TiO_2_) has proven to be a highly efficient material, especially in the field of carbon dioxide reduction and photoelectrochemical water splitting for green hydrogen production.^1−4^ Over the past decade, the interest in B-TiO_2_ has steadily increased in the photocatalytic community.^5−8^ To date, most of the B-TiO_2_ materials are prepared by hydrogen treatment of anatase TiO_2_ nanoparticles.^9,10^ The resulting core–shell particles possess a nonstoichiometric amorphous outer shell and are highly light-absorbing.^11^ Especially with regard to thin-film sputtering, publications showing an alternative process for the deposition of B-TiO_2_ are rare. Escaliante et al. prepared a multilayered TiO_2_/TiO_2-*x*_/TiO_2_ film.^12^ During the reactive sputtering of TiO_2_, they periodically stopped the oxygen flow to create the substoichiometric TiO_2-*x*_ layers. They showed that their samples exhibited an enhanced photocatalytic response compared with pristine TiO_2_. Another way to produce a B-TiO_2_ thin film is by sputtering pristine TiO_2_ thin films and post-treating the samples after deposition using a hydrogen plasma.^13^ Samples prepared in this way are highly absorptive in the UV–visible range and show good conductivity. By introducing hydrogen directly into the plasma during the sputter process, a uniform B-TiO_2_ can be formed.^14,15^ This leads to an enhanced photoelectrochemical activity compared with pristine TiO_2_ as the optical absorption is increased. Recently, we presented a new hydrogen-free bipolar sputter process for the deposition of B-TiO_2_ thin films.^16,17^ This asymmetric dynamic nature of the process results in layered films that show a very high photoelectrochemical activity when compared to pristine TiO_2_. The inhomogeneous films sputtered this way exhibit alternating crystalline and amorphous phases. Similar structures can be found in core–shell B-TiO_2_ nanoparticles. The physical background as to why this structure is beneficial has already been investigated in detail.^8,18,19^ However, such investigations are lacking for thin-film B-TiO_2_. Due to the similarity in structure, similar reasons for the increased photoelectrochemical activity can be assumed. This investigation is the subject of this article.

In order to investigate the underlying reason for the enhanced photoelectrochemical properties of B-TiO_2_ thin films, the microstructure and properties of the dynamic sputtered B-TiO_2_ thin films are studied in detail. We show how the individual sputter phases of this asymmetric process contribute to the improved properties of the thin film. For this, the dynamic part of the process is transformed into static sputtering, i.e., the substrate is kept at a fixed position in front of the targets instead of being moved. This transformation makes it possible to analyze the different formation phases and investigate their properties. The individual phases are characterized in terms of their electrical, morphological, and photoelectrochemical properties. The results are used to discuss how an alternating structure of the crystalline and amorphous phases leads to an enhanced photoelectrochemical water-splitting ability of the dynamic sample, comparable to the core–shell structure of B-TiO_2_ nanoparticles.

## Methods

### Sample Preparation

Samples were prepared by closed-loop reactive bipolar magnetron sputtering using a Vistaris 600 inline vacuum system (Singulus Technologies AG, Germany). Two cylindrical titanium targets (purity 3 N) with a length of 600 mm and a diameter of 165 mm were utilized. The total power of 8 kW was distributed 75% to the left target and 25% to the right target. As substrates, 10 × 10 and 30 × 30 cm^2^ soda-lime glass was taken. A closed-loop feedback control was applied to keep the oxygen flow at a constant oxygen partial pressure. The substrate temperature was set to 200 °C. In the dynamic process, the carrier on which the substrate is mounted passes the targets several times. Samples from this process type will be termed “dynamic samples” in the course of this article. During the static process, two 30 cm × 30 cm glass substrates, mounted on the carrier, were placed stationary in front of the targets inside the sputtering chamber by omitting the carrier movement during deposition. Samples from this process type will be referred to as “static samples” in the course of this article. The sputtering chamber is 58 cm long, and therefore, the substrates can cover the entire chamber. The sputtering time was set to 15 min. A detailed description of the dynamic process can be found in an earlier study.^16^

### Characterization Methods

The thickness *d* of the produced samples was measured with a Dektak 150 stylus profilometer (Veeco). The sheet resistance *R*_s_ was measured with a Jandel RM3-AR four-point probe station (Jandel Engineering Ltd., U.K.). The measured sheet resistance and thickness were used to calculate the resistivity ρ = *R*_s_•*d*. To analyze the crystal structure, Raman measurements were performed using a Senterra system with a 488 nm laser (Bruker Corporation). High-angle annular dark-field imaging in a scanning transmission electron microscope (HAADF–STEM) and energy-dispersive X-ray spectroscopy (EDX) line scan measurements were performed by an FEI Titan 80/300 G1 transmission electron microscope (Field Electron and Ion Company). Electrochemical characterization of the materials was performed with a VersaSTAT 4 potentiostat (Ametek Scientific Instruments). Linear sweep voltammetry (LSV) was performed in a three-electrode setup with a Ag/AgCl reference electrode and a platinum wire as a counter electrode (surface area of 83.68 cm^2^). The deposited B-TiO_2_ layers served as the working electrode with an active area of 0.785 cm^2^. A 1 M KOH solution (pH = 13) was taken as the electrolyte. The scan rate of the LSV measurement was 10 mV/s. A solar simulator with AM 1.5G (1000 W/m^2^) was used for visible-light irradiation.

## Results and Discussion

**Figure 1:**
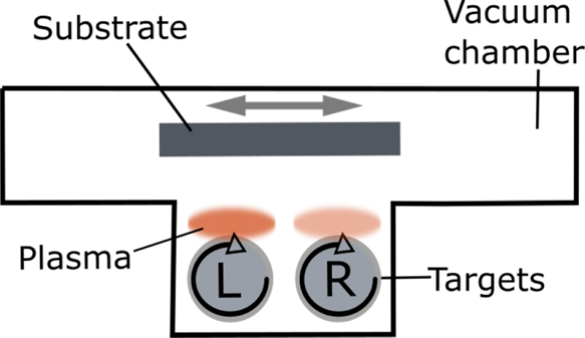
Schematic drawing of the sputter chamber. Inside the vacuum chamber, the substrate moves in front of the two rotary targets, left (L) and right (R). To emphasize the higher power of the left target, the plasma is colored darker.

Due to the moving substrate, the dynamic asymmetric sputter process by nature leads to an inhomogeneous growth of B-TiO_2_ thin films. In the vacuum chamber, the glass substrate first moves along the left target and then the right target; see Figure 1. After passing both targets, the direction of the substrate changes and it moves from the right to the left. This process was repeated seven times.

**Figure 2:**
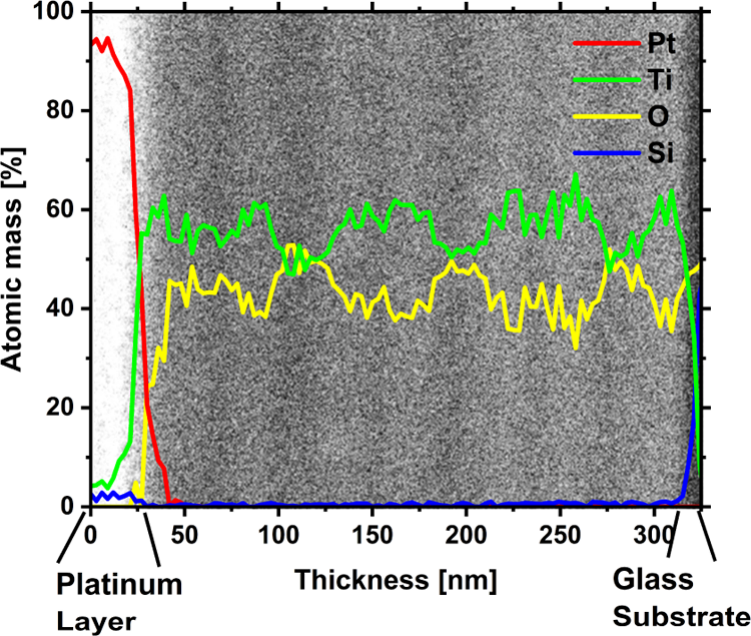
HAADF–STEM image and EDX line scan of a cross section of the sputtered B-TiO_2_ thin film. The platinum protection layer and the glass substrate can be seen on the left and right edges, respectively. The growth direction of the layer is from the right to the left.

An EDX line scan was performed to measure the distribution of titanium and oxygen atoms in the dynamic sample, and the results can be seen in Figure 2. A HAADF–STEM image of a cross section of the thin film is also shown.

The EDX measurement in Figure 2 shows the titanium (Ti) and oxygen (O) distributions as well as the platinum (Pt) and silicon (Si) distributions in the cross section of the dynamic sample. It can be clearly seen that there are alternating areas of higher oxygen and lower titanium contents. In addition, the line scan shows the glass substrate on the right and the protective platinum layer on the left side. The alternating areas can also be differentiated through the different contrast areas in the HAADF–STEM image. This structure can be explained by two different aspects of the process: first, by the dynamic character of the sputter process, and second, by the asymmetric power distribution of the targets. The latter has the effect that the oxidation state, i.e., the composition of the sputtered material, differs between the left and the right target. Since the sample moves back and forth seven times in front of the targets, an alternating structure can be expected. This is confirmed by the EDX data and the STEM image. Further details about the process can be found elsewhere.^16^

**Figure 3:**
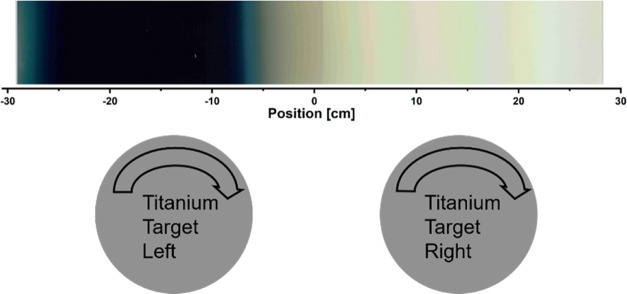
Stationary growth: two glass substrates are fixed in front of the two rotating targets. The dimensions and positions of the targets correspond to the positions of the sample.

In order to gain a deeper understanding of the B-TiO_2_ thin-film growth, static sputtering experiments were performed, i.e., the glass substrates were placed in front of the targets and the movement of the carrier was omitted. In this way, it is possible to record the local growth conditions at each position in the chamber. Figure 3 shows a section of the overall static sample. In addition, the positions of the two targets in relation to the static sample are depicted schematically. For better visualization, an image section of the static samples is shown.

The lateral positions of the centers of rotation of the two titanium targets are at −13 and +13 cm in front of the sample. The targets were operated at different powers: 6 kW on the left and 2 kW on the right. Furthermore, Figure 3 shows that a black layer grows in front of the left target, while the layer that grows in front of the right target appears transparent.

To obtain more information about the growth process, the thickness of the static sample was measured at different positions. In addition, a Gaussian fit was performed on the two peaks of the deposition rate. With this, the growth of the dynamic sample for seven layers can be calculated.

**Figure 4:**
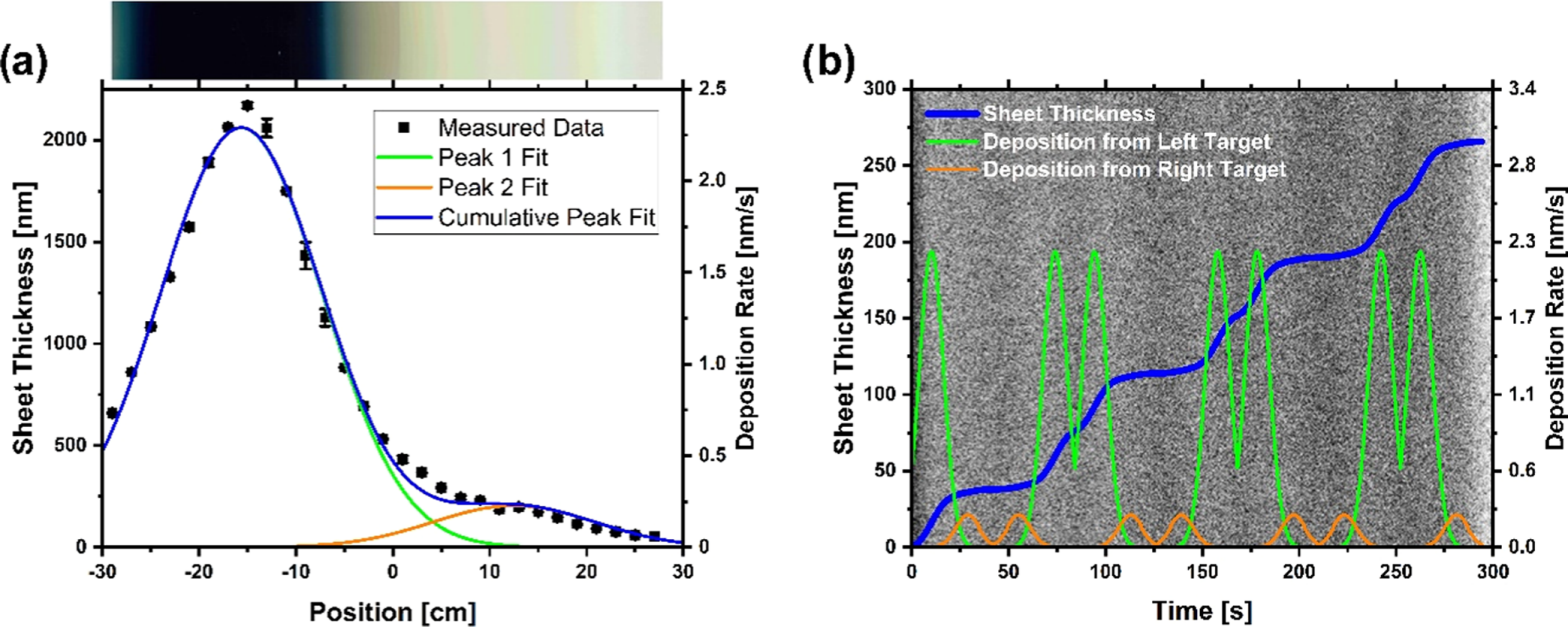
Layer growth analysis of the static and dynamic samples. (a) Measured thickness and corresponding deposition rate of the static sample for different positions. A Gaussian peak fit is included for each of the two targets. For a better visualization of the position, an image of the sample is shown above the graph. (b) From the fits, the layer thickness growths of the dynamic sample over time were calculated. For a better visualization, the fitted deposition rates of both targets are shown as well. In the background, the HAADF–STEM image from Figure 1 can be seen. Here, the glass substrate is on the left side and the platinum layer is on the right side.

Figure 4a shows the measured thickness and the corresponding deposition rate of the static sample with respect to the position inside the chamber. To the left of −29 cm and to the right of 29 cm, the chamber wall blocks further deposition. The deposition rate in the black area is higher than that in the other parts of the sample. The fitted deposition rates are used to calculate the layer growth of the dynamic sample as a function of time; see Figure 4b. It can clearly be seen that the layer grows most during the periods in front of the left target. In addition, the dark areas of the HAADF–STEM image correspond well to periods of a lower deposition rate. These areas also have a lower titanium–oxygen ratio than the brighter areas, as indicated by the EDX measurements in Figure 2. To analyze how this structure affects the electrical properties, resistance measurements were taken at 1 cm intervals over the entire static sample. The resistivity of the dynamic sample was also determined.

**Figure 5:**
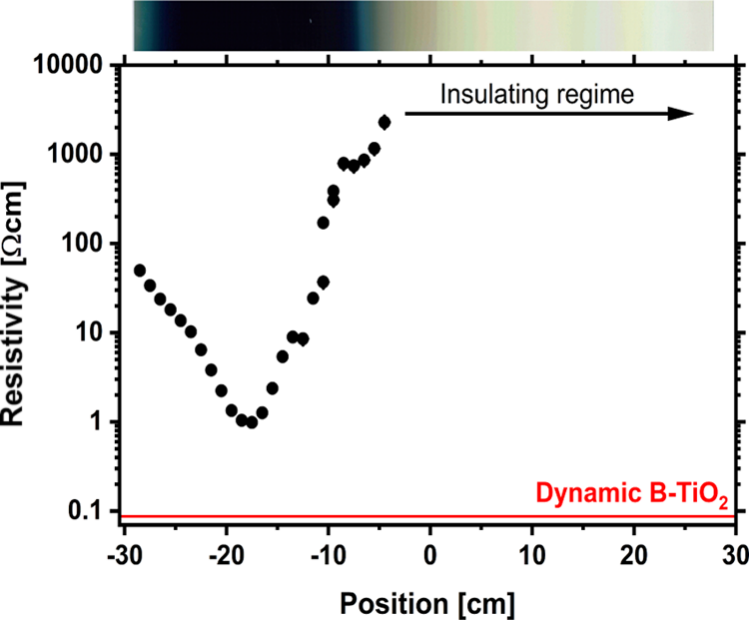
Resistivity measurements of the static sample at different positions. The inset red line shows the measured resistivity of the dynamic sample. For a better illustration, an image of the static sample is shown at the top of the graph.

Figure 5 shows the resistivity measured with a four-point probe, at different positions along the static sample. The point with the lowest resistivity corresponds well with the center of the black area. For positions to the right of −5 cm, the resistivity was too high to be measured. Interestingly, the resistivity of the 250 nm dynamic sample (red solid line in Figure 5) is 1 order of magnitude lower than that of the static sample.

**Figure 6:**
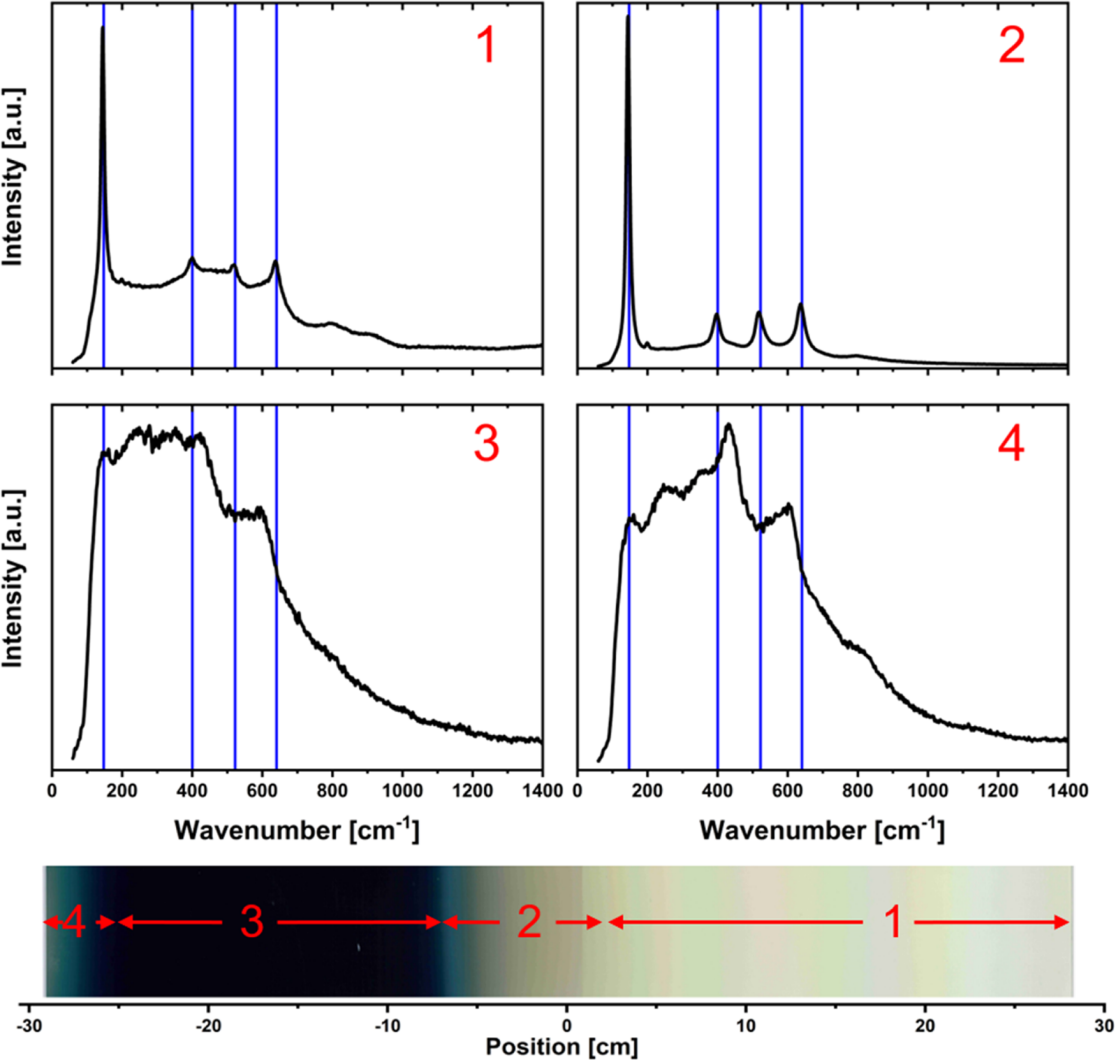
Raman spectra for the four selected positions of the static sample. The numbers represent the corresponding position on the sample. The positions can be seen in the image of the sample. The arrows symbolize the range in which the Raman signal is comparable. The blue vertical lines show the wavenumbers for anatase TiO_2_ peaks (147, 400, 522, and 640 cm^–1^).

To analyze the structural composition of the static sample, Raman measurements were performed for each centimeter of the static sample. A compilation of all Raman spectra can be found in the Supporting Information (S-Gif1). Figure 6 shows the Raman spectra for four selected positions that are representative of their surrounding areas.

The four points of interest are located in front of the right target (1), between the two targets in the transparent area (2), in the center of the black area (3), and to the left of the black area (4). Spectrum 1 shows peaks corresponding to anatase TiO_2_ structures, while at the same time, an amorphous background was observed. Between the two targets (spectrum 2), the anatase peaks are more easily distinguished from the amorphous background. The sample is also thicker than at point 1. In the black area (spectrum 3), only the anatase peak at 147 cm^–1^ remains distinguishable. The amorphous background here is very dominant. However, to the left of the black area (spectrum 4), new peaks appear next to the 147 cm^–1^ anatase peak. These peaks are at 247, 435, and 602 cm^–1^ and cannot be assigned to the anatase phase of TiO_2_. However, due to the broad nature of the peaks, the possible existence of several other crystal structures cannot be excluded. As can be seen from the EDX measurements shown in Figure 2, the region studied exhibits a reduced chemical phase identified as the Magneli phase (Ti*_n_*O_2-*n*_, where *n* is an integer). The exact enumeration of these phases remains elusive, although preparations and analyses have been extended up to *n* = 20.^20^ Notable examples include Ti_2_O_3_, Ti_3_O_5_, and Ti_4_O_7_, which have been extensively studied.^21^ These Magneli phases exhibit superior electrical conductivity compared to stoichiometric TiO_2_ and other Magneli phases with higher values of *n*. Magneli phases with *n* ≤ 4 prove to be promising catalysts, exhibiting enhanced catalytic activity, remarkable stability, and resistance to corrosion. Conversely, for *n* > 4, substantial changes in the crystal structures lead to notable shifts in the catalytic properties.^22,23^ Raman studies of Ti_2_O_3_, Ti_3_O_5_, and Ti_4_O_7_ show a variety of peaks for each phase.^24−26^ The congruence in peak positions between these studies and the broad peaks observed in Figure 6 supports the presence of these three Magneli phases in the sample investigated. However, the absence of an identifiable dominant phase necessitates the assumption of a composite mixture consisting of three distinct phases.

X-ray diffraction (XRD) analyses performed on the dynamic sample indicated the absence of a dominant crystal structure, as corroborated by the Raman spectroscopic measurements presented here.^17^

The compilation S-Gif1 in the Supporting Information shows the Raman spectrum for each point of the sample. There it can also be seen that the transition from the gray to the black area is accompanied by a transition from a crystalline to an amorphous structure. The same transition can be seen on the left side of the black area.

**Figure 7:**
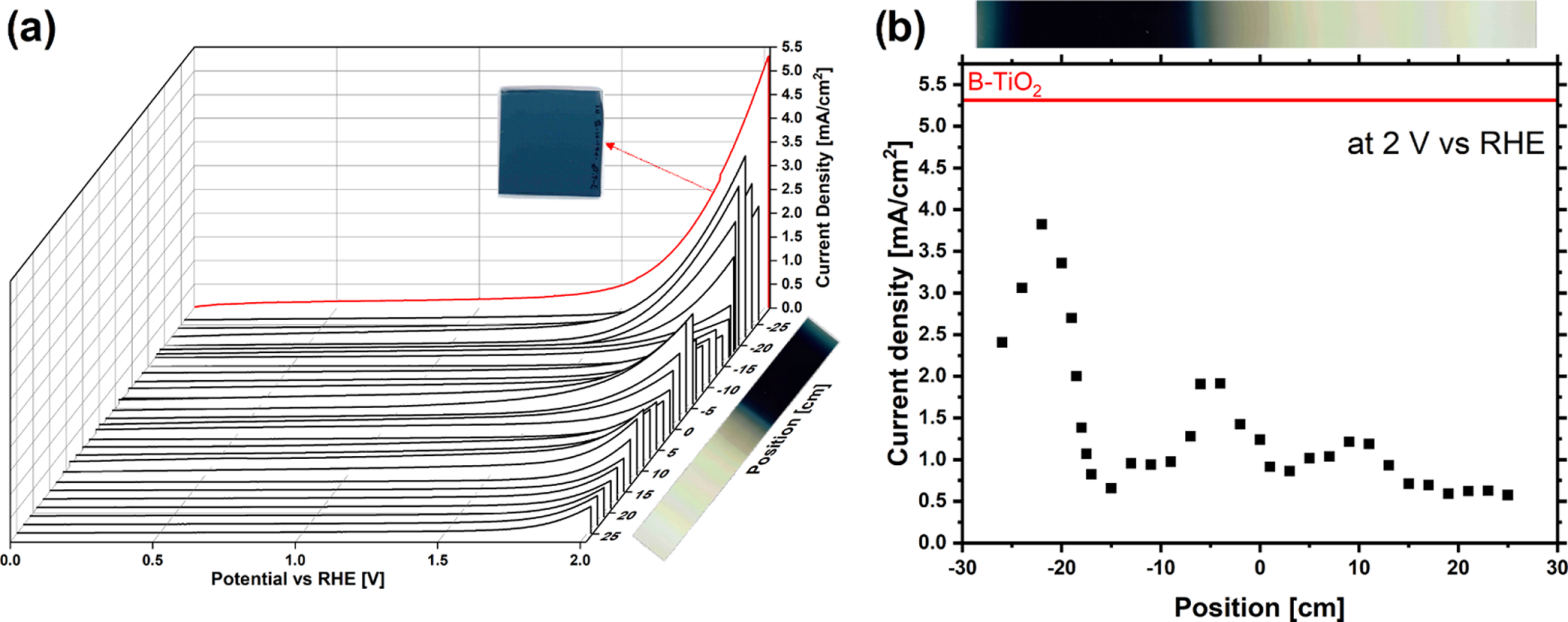
Linear sweep voltammetry. (a) LSV of different positions of the static sample under 1 sun illumination. For better visualization, an image of the sample is shown next to the corresponding positions. The red line shows the LSV measurement for the dynamic sample. Next to the red line, an image of the dynamic sample is shown. (b) Current densities measured by LSV at 2 V vs the reversible hydrogen electrode (RHE) for each centimeter of the static sample. The red line shows the current density of the dynamic sample. For better visualization, an image of the static sample is shown above the graph.

**Figure 8:**
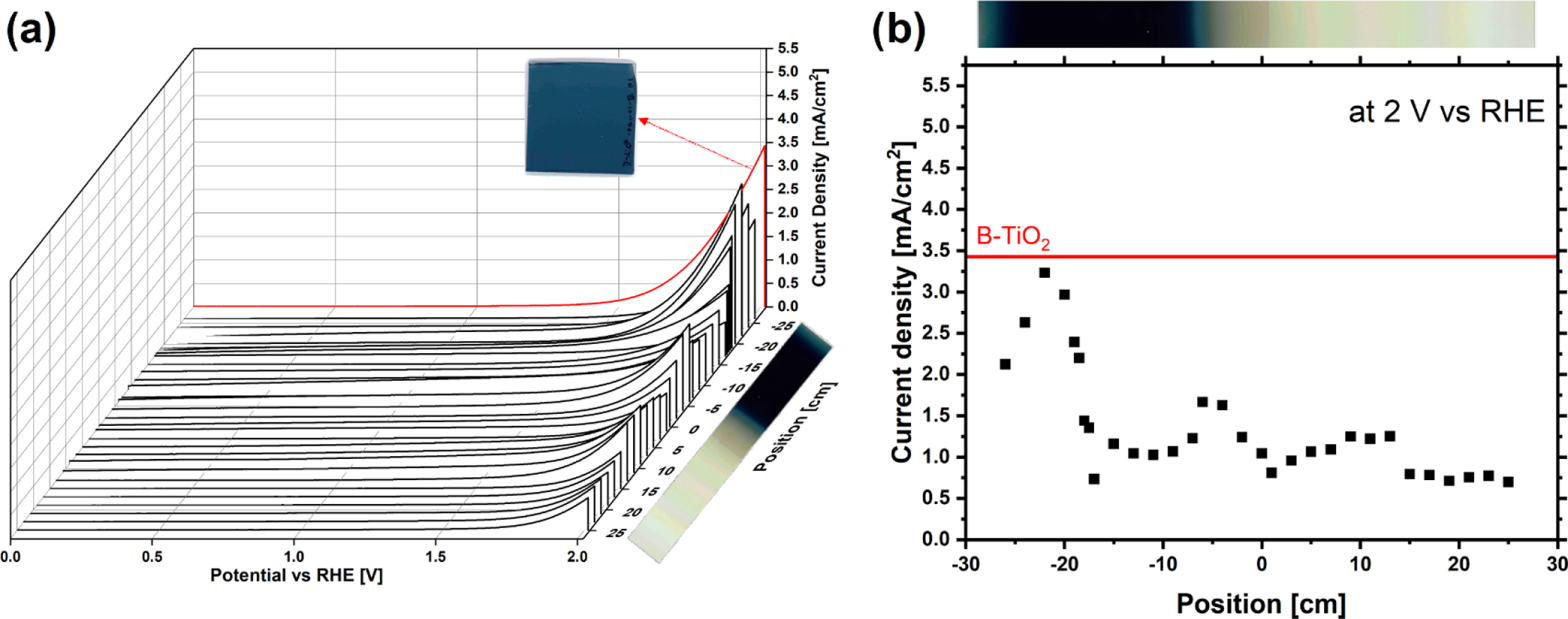
Linear sweep voltammetry without illumination. (a) LSV on different positions of the static sample. For better visualization, an image of the sample is shown next to the corresponding positions. The red line shows the LSV measurement for the dynamic sample. Next to the red line, an image of the dynamic sample is shown. (b) Current densities measured by LSV at 2 V vs RHE for different positions of the static sample. The red line shows the current density for the dynamic sample. An image of the static sample is shown at the top of the graph.

To understand the influence of the single phases toward the enhanced photoelectrochemical activity of the dynamic sample, linear sweep voltammetry (LSV) measurements were done on each centimeter of the static sample as well as on the dynamic sample. The active area was a circle with a 1 cm diameter. In that way, each spot of the static sample can be measured. The measurements at 1 sun and in darkness are shown in Figures 7 and 8, respectively. The position of the measurements shown in the graph corresponds to the center of the active area.

The red curve in Figure 7a represents the LSV for the dynamic sample, and the black lines are the LSV measurements for each centimeter of the static sample. Additionally, Figure 7b shows the current densities measured at 2 V vs RHE. While the dynamic sample (red curves) shows the highest current density, two regions of the static sample also show remarkably high current densities. These two areas are located at the edge of the crystalline and amorphous regions, as shown by the Raman measurements. However, the regions do not have the same maximum photocurrent densities. The photocurrent to the left of the black region is higher. This can be explained by the different crystal structures at these positions. While the Raman spectrum of the region to the right of the black region shows an anatase crystal structure, the position to the left of the black region consists of Ti_3_O_5_ phases. These crystal structures are known for their enhanced photoelectrochemical performance compared to pristine TiO_2_.^27^ However, this does not explain the enhanced photocurrent of the dynamic sample. Lü et al. investigated a similar behavior in nanoparticles of B-TiO_2_.^18^ They studied the interface of the crystalline core with the amorphous shell in order to understand the improved conductivity compared to pristine anatase nanoparticles. To do this, they created TiO_2_ homojunction films consisting of an oxygen-deficient amorphous layer on top. They found that metallic conduction is enabled at the interface, where band bending occurs due to different positions of the Fermi level relative to the conduction band. This leads to an accumulation of electrons at the interface of the crystalline side, resulting in a high interfacial carrier concentration. They conclude that this is the reason for the enhanced electron transport of B-TiO_2_. The Raman measurement shown in this article suggests a similar behavior, which would explain the enhanced conductivity and photocurrent of the dynamic sample.

Figure 8 shows the LSV measurements without illumination. The influence on the photoinduced carriers can be seen here.

The LSV measurements of the static sample in the dark show a pattern similar to the measurements under illumination. Two areas of increased current density are located at the edges of the black region, the same as that for the measurement under illumination. While the current densities observed at the peak positions of −5 and 10 cm show an approximate equivalence between measurements carried out under illuminated and dark conditions, a noticeable divergence appears at the −21 cm position, where the current density significantly exceeds that observed in the dark. This observed phenomenon can be attributed to the presence of several Magneli phases in this region of the sample, which exhibit enhanced photocatalytic activity in contrast to the predominant TiO_2_ phase observed in other regions of the sample. Consequently, the enhanced water-splitting activity catalyzed by the Magneli phases is shown as an increased current density under illumination conditions.

However, as shown in Figure 8b, the current density of the dynamic sample at 2 V vs RHE is not increased compared to the static sample. Moreover, it is in good agreement with the highest current density of the static sample. The difference in current density between illumination and darkness is by far the largest for the dynamic sample. These alternating crystalline and amorphous phases might lead to an enhanced extraction of the photogenerated charge carriers, as explained above. The individual positions on the static sample, especially in the black area, do not have this effect and therefore show a reduced photoelectrochemical behavior.

Direct comparison with other studies is difficult. However, to obtain the results of this work into context, two comparable samples for the literature are taken as exemplary. Liang et al. sputtered an amorphous black TiO_2_:H thin film and achieved a saturated current density of 0.67 mA/cm^2^ at 1.23 V vs RHE with 1 M NaOH as the electrolyte under AM 1.5 irradiation.^15^ Cho et al. created multiple heterojunctions of crystalline anatase/disordered rutile/ordered rutile layers within a single TiO_2_ nanoparticle. Using this method, they achieved a current density of 1 mA/cm^2^ at 1.9 V vs RHE with 1 M KOH as the electrolyte under irradiation of 1 sun.^28^ However, the photocurrent density of 5.26 mA/cm^2^ at 2 V vs RHE of the dynamic sample shown in this work is significantly higher.

## Conclusions

In this work, the influence of the dynamic nature of asymmetric bipolar magnetron sputter processes on the growth of B-TiO_2_ is studied, aiming to understand its enhanced photoelectrochemical activity. In order to study the layered structure of the dynamically sputtered films, static sputtering was used to analyze the individual phases.

It was found that the asymmetric process mainly leads to two distinct phases, a low light-absorbing crystalline phase and a high light-absorbing amorphous phase. We show that the dynamically sputtered sample exhibits superior photoelectrochemical properties compared to any of the individual phases from the static process. Notably, the electrical resistivity decreased by a factor of 10, accompanied by a substantial increase in the measured current during photoelectrochemical water splitting. This enhancement is most likely attributed to an improved extraction of photogenerated charge carriers at the amorphous/crystalline interface. This behavior was also described in the context of B-TiO_2_ core–shell nanoparticles. Consequently, the findings presented in this article mark a significant step toward the development of large-area B-TiO_2_ thin films with enhanced capabilities for photoelectrochemical water splitting, thereby fostering progress in sustainable energy technologies.

## Supporting Information Available

Compilation of all Raman spectra (MP4)
